# Systemic Administration of Tranexamic Acid Improves Postoperative Outcome in Abdominoplasty

**DOI:** 10.3390/jcm14217556

**Published:** 2025-10-24

**Authors:** Leila Sahinovic, Marie Louise Kohne, Jun Jiang, Hans-Guenther Machens, Haydar Kükrek, Ulf Dornseifer, Daniel Schmauss, Philipp Moog

**Affiliations:** 1 Department of Plastic Surgery and Hand Surgery, Klinikum Rechts der Isar, Technische Universität München, Ismaninger Str. 22, 81675 Munich, Germany; leila.sahinovic@icloud.com (L.S.); marykohne@web.de (M.L.K.); junqing.jiang@mri.tum.de (J.J.); hans-guenther.machens@mri.tum.de (H.-G.M.); haydar.kuekrek@mri.tum.de (H.K.); ulf.dornseifer@isarklinikum.de (U.D.); schmauss.daniel@gmail.com (D.S.); 2 Department of Plastic Surgery and Hand Surgery, Kepler Universitätsklinikum, 4021 Linz, Austria; 3 Department of Plastic, Reconstructive and Aesthetic Surgery, Isar Klinikum, 80331 Munich, Germany; 4 Department of Plastic, Reconstructive and Aesthetic Surgery, Ospedale Regionale di Lugano, Ente Ospedaliero Cantonale (EOC), 6900 Lugano, Switzerland; 5 Faculty of Biomedical Sciences, Università della Svizzera Italiana (USI), 6900 Lugano, Switzerland

**Keywords:** tranexamic acid, TXA, abdominoplasty, body contouring, plastic surgery

## Abstract

**Background/Objectives**: In plastic surgery, the administration of tranexamic acid (TXA) has gained increasing support by current literature, highlighting its relevance in clinical practice. This study evaluates the perioperative impact of prophylactic intravenous TXA administration in abdominoplasty, focusing on surgical outcome, drainage pattern, complications, and laboratory parameters (hematocrit/hemoglobin). **Methods**: This retrospective, single-center cohort study analyzed 58 abdominoplasties, which were divided into two groups: patients treated perioperatively with tranexamic acid for 48 h (TXA group; *n* = 24) and without TXA (no-TXA group; *n* = 34). **Results**: Patients in the TXA group had a significantly shorter length of hospital stay (*p* = 0.008) and a lower postoperative daily drainage volume on postoperative days: 3 (*p* = 0.047), 4 (*p* = 0.011), 7 (*p* = 0.014), 8 (*p* = 0.024), and 9 (*p* = 0.042). The overall complication rate was also significantly reduced with TXA (25.0% vs. 52.9% in the no-TXA group; *p* = 0.033). Postoperative declines in hematocrit and hemoglobin were less pronounced in the TXA group (*p* = 0.353 and *p* = 0.255, respectively). Furthermore, the intravenous administration of TXA was well tolerated, and no associated thromboembolic events were observed. **Conclusions**: Intravenous TXA appears to reduce complications, drainage volumes, and hospital stay in abdominoplasty patients, while being safe and well tolerated. Although further studies are needed to define optimal dosing, administration protocols, and long-term safety, these findings support the potential benefits of TXA for both patients and healthcare systems, thereby enabling a standardized approach to body contouring surgery.

## 1. Introduction

Body contouring surgery has advanced considerably over the past decade, largely due to the increasing number of patients following massive weight loss and the development of innovative fat reduction techniques [1]. In 2024, a total of 626,215 esthetic procedures were recorded in Germany [2].

Abdominoplasty is one of the most popular and most frequently performed procedures in plastic surgery, accounting for 6.2% of all procedures [2]. Despite increasing safety and surgical advances, this operation still has a comparatively high complication rate of up to 43% [3,4]. The most common complications are hematoma and seroma formation, as well as wound dehiscence and infection [1].

In the context of esthetic surgery, potential complications are particularly feared, as the patients usually undergo elective operations. Bleeding complications, the need for revision surgery or blood transfusions can severely impair the postoperative result and outcome for the patient, while also incurring additional costs [5].

Tranexamic acid (TXA) is a synthetic lysine analog antifibrinolytic agent that competitively inhibits the activation of plasminogen to plasmin [6]. In high concentrations, it blocks plasmin non-competitively, meaning that TXA inhibits the dissolution and degradation of fibrin clots by plasmin [6].

Numerous studies in the fields of orthopedics, traumatology, and obstetrics have already demonstrated that the administration of TXA reduces postoperative bleeding without increasing significant thromboembolic events [7,8,9]. The use of TXA is considered safe and well tolerated [10]. In plastic surgery, the administration of TXA is increasingly supported by studies and is gaining relevance for clinical practice [11,12,13]. A reduction in the postoperative seroma rate, drainage volume, as well as a reduced hemoglobin decline after intravenous administration of TXA following abdominoplasty has been demonstrated [12,14]. In addition, a reduction in daily drainage volume and thus in situ retention of drains was demonstrated with topical TXA application [11,13]. In breast surgery, systemic TXA use has been linked to decreased drainage volume, shorter hospital stays, and, in some cases, reduced postoperative bleeding [13,15].

Nevertheless, the perioperative administration of TXA to reduce perioperative bleeding is not yet standardized and is considered off-label use [6]. The objective of this comparative study is to examine the perioperative effects of prophylactic systemic administration of TXA following abdominoplasty, with reference to its impact on surgical outcome, drainage pattern, complications, and laboratory parameters (hematocrit/hemoglobin) in particular. The off-label use of TXA is intended to optimize and ensure the safety of surgical treatment, thereby increasing patient safety in general.

## 2. Materials and Methods

### 2.1. Ethics

This study was approved by the relevant ethics committee (reference number: 250-370-S-CB; date of approval: 6 August 2025). This study was conducted in accordance with the principles of the Declaration of Helsinki and local ethical guidelines. Due to the retrospective study design, it was not possible to obtain active consent from the participants retrospectively.

### 2.2. Study Design

This retrospective, single-center cohort study included all patients who underwent abdominoplasty between January 2020 and June 2025 (=66 months) and who were treated with tranexamic acid (TXA group; *n* = 24) or did not receive it (non-TXA group; *n* = 34) perioperatively. The analysis is based on routine clinical data collected from the study center’s electronic and analog patient records. There were no retrospective patient exclusions.

Demographic and clinical parameters such as age, body mass index (BMI), and gender at the time of surgery were recorded. In addition, relevant pre-existing conditions, comorbidities, and medication were highlighted, the duration of postoperative follow-up care was considered, and a distinction was made according to indication (esthetic, post-bariatric). In addition, surgery-specific parameters were analyzed. These included the type/technique of surgery, combined procedures, resection weight (grams), the number of drains used and the duration of surgery (incision–suture time in minutes). Combined procedures were defined as abdominoplasties performed in conjunction with breast procedures (mastopexy, breast augmentation with autologous fat transfer or breast augmentation using implants). Additional liposuction was only performed in combination with abdominoplasty, and therefore was not classified as a combined procedure. For the analysis of surgical duration, combined procedures and isolated abdominoplasties were assessed individually.

Primary outcome measures were defined as length of hospital stay (in days), postoperative drainage pattern, as well as the occurrence of postoperative complications, in particular seroma formation, wound healing disorders, and the need for blood transfusion (packed red blood cells). Postoperative complications were reported in a standardized manner as minor and major complications according to Clavien–Dindo, as already established in other studies [16,17]. Drainage pattern was analyzed based on daily output (mL), total output (mL), and the duration of drain in situ (days). The evaluation was limited to drains in the abdominal region.

Secondary endpoints included hemodynamic changes (hematocrit and hemoglobin) pre- and postoperatively. A 60-day postoperative follow-up period was implemented, to ensure that only surgery-related complications were included.

### 2.3. Surgical and Perioperative Procedures

All included procedures were performed in a standardized manner and under general anesthesia. The incision, dissection, rectus diastasis repair, liposuction, and skin/fat apron resection were performed depending on the indication in the form of a conventional abdominoplasty with umbilical transposition or fleur-de-lis abdominoplasty. In 12 patients, additional breast surgery was performed and thus classified as a combined procedure. Redon drains were inserted in all cases and separately removed once the drainage volume was less than 30 mL in 24 h. Contraindications include known hypersensitivity to the active ingredient, previous thromboembolic events, coagulation disorder, subarachnoid hemorrhage, severe renal insufficiency, a history of seizures, and the simultaneous use of combined hormonal contraceptives [18]. After ruling out any contraindications, TXA was used with an intravenous dose of 1 g tranexamic acid intraoperatively at the time of the incision and administered 1-1-1 for 48 h during the inpatient stay, at the discretion of the responsible plastic surgeon. The TXA regimen was chosen based on the current literature [7,8,9,19]. Patients who were discharged on the first postoperative day were switched to oral administration to ensure that a 48 h course of treatment was completed in the same dosage. The follow-up treatment for all patients was standardized, including compression therapy.

### 2.4. Statistical Analysis

All statistical analyses were performed using SPSS software (version 30.0.0.0, manufacturer: IBM Corp., Armonk, NY, USA). Descriptive methods were used to present the basic characteristics (e.g., age, gender, BMI, duration of surgery). Arithmetic mean, standard deviation, median, minimum, and maximum were calculated. The comparison of both groups (TXA vs. no-TXA) was performed depending on the distribution pattern for normally distributed variables using the t-test for independent samples or the Mann–Whitney U test for non-normally distributed variables. The decision on the test selection was based on the Shapiro–Wilk test to check for normal distribution. Categorical variables such as gender, the presence of comorbidities, or the occurrence of minor and major complications were compared using the chi-square test. In comparison groups with *n* ≤ 5 cases, Fisher’s exact test was applied. The log-rank test was used to describe the Kaplan–Meier estimate. A *p*-value < 0.05 was defined as statistically significant.

## 3. Results

### 3.1. Demographic Data

A total of 58 patients were included in the study, comprising 24 in the TXA group and 34 in the no-TXA group (Table 1). The demographic data of the two study groups showed no significant differences between the groups in terms of age (*p* = 0.363), BMI (*p* = 0.145), gender (*p* = 0.726), comorbidities such as diabetes (*p* = 0.499), nicotine use (*p* = 1.000), anticoagulation (*p* = 0.134), or thromboembolic history (*p* = 0.260). The follow-up period was set to 60 days post intervention.

### 3.2. Surgery-Specific Data

The indication for abdominoplasty in the overall group was predominantly post-bariatric (69.0%), followed by esthetic procedures (31.0%). When the indications were differentiated between the TXA and non-TXA groups, the procedure was performed more frequently as an esthetic procedure in the TXA group (45.8% vs. 20.6%), while post-bariatric indications dominated in the no-TXA group (79.48% vs. 54.2%, *p* = 0.041) (Table 2).

There was no significant difference between the two groups in terms of the surgical techniques used (conventional abdominoplasty, fleur-de-lis) (*p* = 0.984). Additional liposuction was performed in 29.3% of all patients, with this being more frequently performed in the no-TXA group (35.3% vs. 20.8%, *p* = 0.260). 20.7% of patients underwent combined procedures, with comparable distribution between two groups (25.0% vs. 17.6%; *p* = 0.496). There was a difference between the two groups in terms of rectus diastasis repair being more often performed in the TXA group (62.5% vs. 26.5%; *p* = 0.006). In addition, the mean resection weight was lower in the TXA group (1713 g ± 1846 g) than in the no-TXA group (2438 g ± 1676 g; *p* = 0.024). Time of incision to suture is presented separately for abdominoplasty only and for abdominoplasty with combined procedures. There was no significant difference in the duration of surgery between the two groups (abdominoplasty only: TXA: 3:11 ± 0:40 h; no-TXA group: 3:24 ± 0:43 h; *p* = 0.338; combined procedures: TXA: 5:55 ± 0:55 h; no-TXA group: 5:30 ± 1:43 h; *p* = 0.512). Overall, no significant difference regarding the primary and secondary endpoints was detected between patients who underwent combined procedures or abdominoplasty only.

### 3.3. Postoperative Outcome and Drainage Pattern

**Figure 1:**
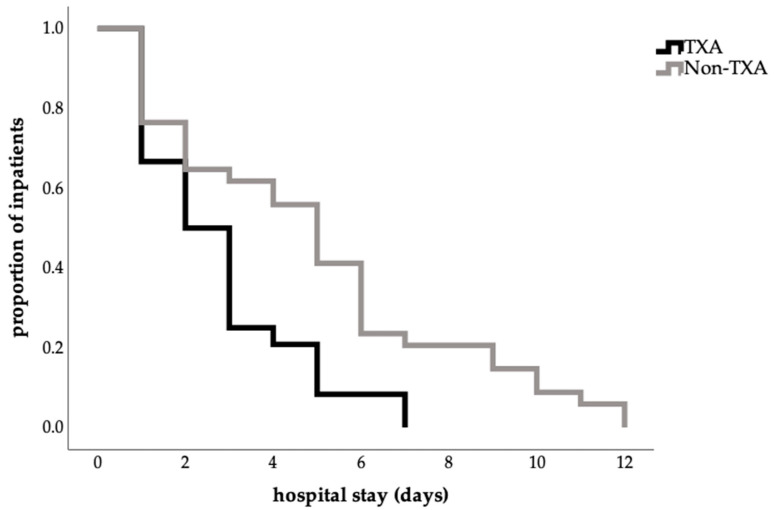
Influence of TXA on length of hospital stay (Kaplan–Meier estimate of hospital stay in days, proportion 1.0 = 100%percent; log-rank test, *p* = 0.004).

The length of hospital stay was shorter in the TXA group (2.79 ± 1.86) than in the no-TXA group (4.94 ± 3.08), corresponding to a mean reduction of 2.2 days (*p* = 0.008; Table 3). This finding was also confirmed by the log-rank test (*p* = 0.004; Figure 1).

The mean time to total drain removal was relatively shorter in the TXA group (3.85 ± 1.87 days vs. 6.00 ± 4.97 days); however, this difference did not reach statistical significance (*p* = 0.243; Table 3). The time to first drain removal tended to be similar in both groups (TXA: 2.00 ± 0.95 days; no-TXA group: 2.17 ± 1.28 days; *p* = 0.729; (Table 3)).

**Figure 2:**
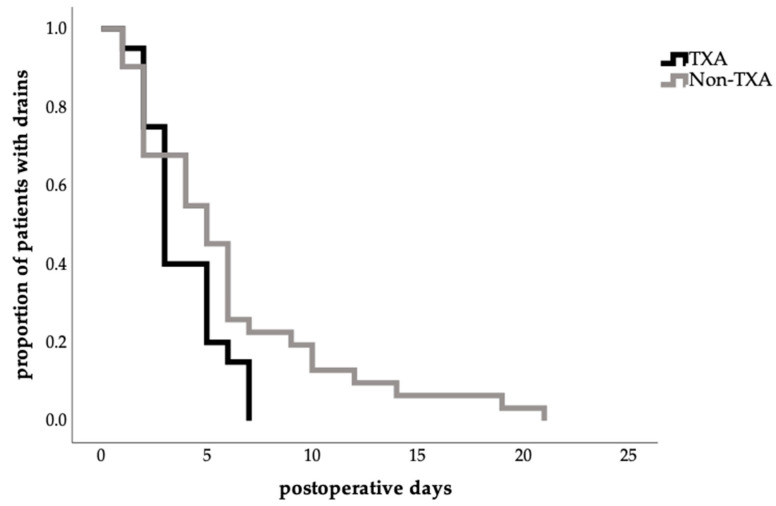
Effect of TXA on time to total abdominal drain removal (Kaplan–Meier estimate time to drain removal in days, proportion of patients with drains in percent 1.0 = 100%; log-rank test, *p* = 0.065).

Notably, total drain removal in the TXA group occurred more frequently between postoperative days 3 and 5 in the TXA group, with no retention beyond 7 days, whereas prolonged drain durations of up to 21 days were observed in the no-TXA group (Figure 2).

Although the mean total drainage volume was lower in the TXA group (292 ± 255 mL) compared to the no-TXA group (614 ± 712 mL), this difference was not statistically significant (*p* = 0.352; Table 3).

**Figure 3:**
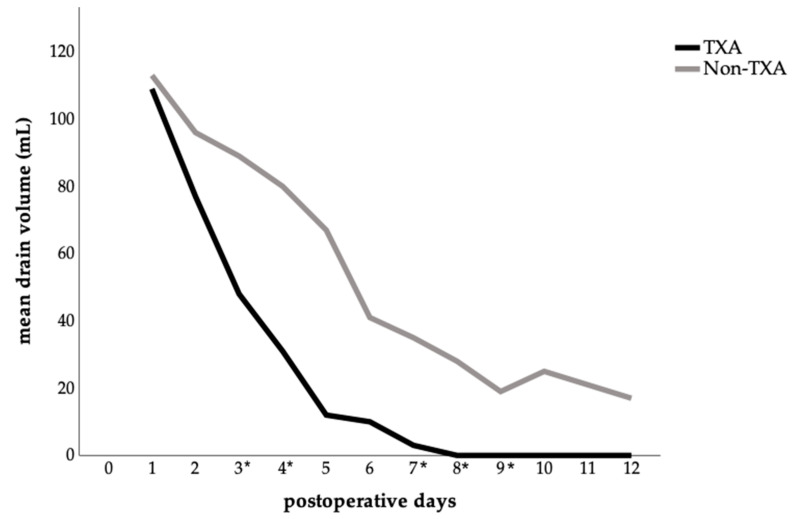
Postoperative abdominal drainage volume comparing the TXA and non-TXA group. The data are presented as the mean value for each postoperative day. Statistical significance was assessed by comparing the daily mean values between groups using either the t-test or the Mann–Whitney U test, depending on the distribution pattern; (*p* < 0.05) is marked with an (*) on the *x*-axis.

A detailed analysis of mean abdominal drainage volume per postoperative day showed clear differences between the two groups. In the TXA group, drainage volumes declined rapidly, whereas in the control group, they remained higher and more persistent (Figure 3). Significant reductions in daily drainage output were observed in the TXA group on postoperative days: 3 (*p* = 0.047), 4 (*p* = 0.011), 7 (*p* = 0.014), 8 (*p* = 0.024), and 9 (*p* = 0.042), compared with the no-TXA group.

### 3.4. Complications

The total rate of postoperative complications (minor and major complications) was significantly lower in the TXA group (TXA: 25.0%, no-TXA group: 52.9%, *p* = 0.033). When analyzed separately, both minor and major complications were seen more often in the no-TXA group (*p* = 0.356, *p* = 0.171, respectively).

Overall, postoperative seromas occurred in 14 patients (24.1%), being less frequently seen in the TXA group (17.4%) compared to the no-TXA group (29,4%), although this did not reach statistical significance (*p* = 0.361). Four of these seroma formations were classified as major complications due to the need for surgical intervention (three replacement of drains and one seroma evacuation).

Wound healing disorders were observed less frequently in the TXA group (12.5%) compared to the no-TXA group (29.4%) which was not significant (*p* = 0.202). The need for blood transfusion showed no difference between the two groups (TXA: 4.0%; no-TXA group: 6%; *p* = 1.000) (Table 4).

### 3.5. Laboratory Parameters

Postoperatively, the decrease in hematocrit (hct) and hemoglobin (hb) was relatively lower in the TXA group (hct decrease: 7.66 ± 3.20%; hb decrease: 2.47 ± 1.08 g/dl) than in the no-TXA group (hct decrease: 8.55 ± 3.52)%; *p* = 0.353; hb decrease: 2.82 ± 1.09 g/dl; *p* = 0.255; Table 5), but this was not significant.

## 4. Discussion

Achieving a low complication rate in surgical disciplines is the leading factor to ensure improved patient safety and high quality of care and thus to optimize the postoperative outcome of the patient. This study showed that the administration of TXA in patients undergoing abdominoplasty was associated with a shorter length of stay (Figure 1, Table 3), and a faster drain removal in total, being more frequent between the third and fifth postoperative day (Figure 2, Table 3). Furthermore, a significant reduction in postoperative drainage volume from the third postoperative day onwards (Figure 3) and a lower complication rate (Table 4) have also been demonstrated. These results confirm findings from other surgical disciplines such as orthopedics, cardiothoracic surgery, and gynecology, where TXA has been used successfully for years and is established in guidelines [7,8,9,20,21].

Despite the absence of guidelines in plastic surgery, studies have shown an increasing relevance towards TXA administration and thus enhance its relevance for clinical practice [11,12,13]. The indication for topical application, as well as for intravenous administration, is gaining importance in body contouring and breast surgery [14,22,23].

To date, no guidelines for TXA in the field of plastic surgery exist and, thus, our results confirm previous findings regarding the benefits of its efficacy in terms of decreased postoperative complications such as seroma formation or bleeding [11,12,13,14,15].

The basic characteristics of the groups showed comparable patient demographics (Table 1). However, the cohort is heterogeneous in terms of indications and surgical procedures, which reduced the comparability and external validity of the results (Table 2).

In particular, the distribution of surgical indications was uneven, with a higher proportion of esthetic procedures in the TXA group (46% vs. 21%). This discrepancy may have introduced selection bias and thereby limited the generalizability of the findings. To address this limitation, future prospective studies with randomized or matched patient allocation are needed to better control this potential confounder. Ideally, esthetic and post-bariatric patient cohorts should be analyzed separately, given their distinct clinical characteristics and perioperative courses.

A few studies show that a higher resection weight in abdominoplasty is associated with increased drainage volumes, longer drain duration, and increased seroma rates [24,25]. Therefore, a relevant confounder in our study was the significantly higher resection weight observed in the no tranexamic acid (TXA) group, which is attributable to the higher proportion of post-bariatric procedures within this cohort. This distinction is critical, as bariatric surgery induces substantial micro- and macroscopic alterations in subcutaneous adipose tissue, leading to compromised tissue quality and an increased risk of postoperative complications such as seroma formation [26]. Moreover, distinct risk factors that elevate the likelihood of complications, including impaired wound healing and serous fluid collections, have been shown among post-bariatric patients undergoing procedures such as abdominoplasty [27].

In our study, we were able to demonstrate a significantly shorter length of stay in the TXA group (Table 3, Figure 1), as well as a significant reduction in drainage volume from the third postoperative day onwards (Figure 3). This finding, however, must be interpreted with caution. Beyond clinical differences, non-clinical factors—such as financial incentives for earlier discharge of patients undergoing esthetic procedures—may have further influenced this outcome and should be considered a limitation of the present study. Since a substantial proportion of patients with esthetic indications were discharged on the first postoperative day and the TXA group included more esthetic procedures, this may have contributed to the shorter hospital stay observed in this group. Despite this, our results are largely consistent with the current literature [11,12,15]. Other retrospective analyses have shown a significant reduction in length of stay as well as a reduction in drainage volume and duration of drain for both intravenous and topical application, with no evidence of increased complication rates [11,14]. A recent study showed no significant difference in drainage volume between the TXA and non-TXA group but a significantly decreased median drain duration in the TXA group [12]. In the field of breast reduction surgery, two studies, likewise, reported a significant reduction in drainage volume and length of stay [15,23].

A previous study demonstrated that a single topical application of TXA can reduce drainage volume by 54% [11] compared with 41,3% in our study and time of drain removal by 23% [11] in comparison to 25.4% in our cohort, resulting in a shorter hospital stay [11]. Reported reductions in length of stay range from 21.4% to 24% [11,14], whereas a reduction of 33.6% was shown in our study. Other studies, by contrast, found no significant difference in total drain output, although they did report earlier drain removal, with a median duration of 4 days [12] compared with a mean of 3.85 days in our cohort. Comparable results between intravenous and local application have also been described [14,15,23].

A recent meta-analysis on the use of TXA in body contouring surgery consistently reported a reduction in drain output, drain duration, and a shorter hospital stay under TXA across several studies [28]. It also demonstrated a significant reduction in hematoma formation and a trend towards a lower risk of infection, but no proven benefit of TXA regarding seroma prevention. These findings support the assumption that reduced drainage volume using TXA leads to earlier drain removal and thereby shortens hospital stay.

In our findings, the complication rate, in total with 41.4%, is comparable to the current literature [3,4]. The overall rate of postoperative complications was significantly lower in the TXA group, with 25% (Table 4) aligning with current studies that describe reductions in postoperative seromas and hematomas following TXA administration [12,14,29]. Seroma formation or wound healing disorders typically occur within the early postoperative phase, and can therefore be reliably detected with our follow-up period [30,31].

However, the analysis of these complications individually did not show any significant difference in our study. In contrast to that, a lower rate of postoperative bleeding when administered systemically was shown following breast reduction [13], while another study reported increased postoperative bleeding in the TXA group [12].

On the other hand, no complications in the TXA group occurred due to the administration itself, which was also demonstrated in recent studies [11,12,14]. In our TXA cohort, the intravenous administration was well tolerated and safe, as shown in previous studies [10,11,15].

Furthermore, the surgical technique represents as a potential confounder. Multiple analyses show robust evidence that operative techniques such as progressive tension sutures (PTS) significantly reduce the postoperative seroma rate and operative revision following abdominoplasty [32]. PTS were performed inconsistently, according to the surgeon’s preference, which can be considered a bias. In addition to that, the higher rate of rectus diastasis repair in the TXA group may have acted as a relevant confounder potentially influencing the observed outcomes.

In our study, both hematocrit and hemoglobin decline were relatively lower in the TXA group. (Table 5). Although the difference was not significant, this result is consistent with the findings of other studies, which also tended to observe lower blood loss with TXA [14,22]. It has been shown that the combination of preoperative and continued postoperative administration of TXA reduces the decline in hemoglobin and significantly reduces the transfusion rate [22].

This study has several limitations. As a retrospective, single-center study, neither randomization nor blinding was possible, leaving the risk of systematic bias. The relatively small sample size further limits statistical power. A major source of bias lies in the non-standardized administration of TXA, which was left to the discretion of the operating surgeon. Since procedures were performed by different surgeons with potentially varying criteria for TXA use, selection bias cannot be excluded. This may have influenced the observed differences in outcomes and should be considered a key limitation of the present study. Patients with higher bleeding risk due to comorbidities may have been more likely to receive TXA, while those considered low-risk may have been excluded from treatment. Similarly, patients with a history of thromboembolic events—often presenting with more comorbidities and anticoagulant use—may have been excluded from TXA administration, potentially contributing to a higher complication rate in the no-TXA group. Liposuction was performed more frequently in the no-TXA group. Although not statistically significant, it may have exerted a minor influence on the complication rate. Likewise, 12 patients underwent additional breast surgery, which, although not significantly affecting outcomes in this study, could have introduced further variability.

Overall, despite the limitations mentioned above, our results are promising and provide important information that TXA has clinical benefits in plastic and esthetic surgery, particularly in post-bariatric surgery. Prospective, multicenter, and randomized studies are needed to clarify whether TXA reliably reduces seromas and wound healing disorders beyond its drain efficacy and to additionally compare the different forms of application (intravenous vs. topical vs. combined). Our results suggest that perioperative TXA use in abdominoplasty may reduce hospital stay, postoperative drainage, and complications without increasing thromboembolic risk. These findings support the consideration of TXA administration, especially in patients with an elevated risk of bleeding or seroma formation, such as those undergoing extensive tissue resections or with altered tissue quality (e.g., post-bariatric patients). However, additional research is required to confirm our findings and further clarify the efficacy of TXA in reducing postoperative complications such as seroma formation and bleeding. Larger prospective studies are warranted to establish clear recommendations for TXA use in body contouring procedures and to identify the patient populations that benefit the most.

## 5. Conclusions

Achieving a low complication rate is paramount in surgical disciplines to ensure improved patient safety, high quality of care, and optimal postoperative outcomes. The use of tranexamic acid (TXA) has gained increasing importance, particularly in body contouring surgery. Our study demonstrated that intravenous TXA administration during abdominoplasty was associated with a significant reduction in postoperative daily drainage volume, shorter inpatient stays, and a lower overall complication rate without any observed thromboembolic events. Notably, the postoperative complication rate was significantly lower in the TXA group compared to the non-TXA group. Nevertheless, further randomized controlled trials are needed to confirm these findings, evaluate optimal dosing regimens, and establish standardized protocols for TXA use in body contouring procedures.

## Figures and Tables

**Table 1:** Demographic data. Results are given as number (percentage), mean (standard deviation) and [minimum|maximum]. *p*-values are calculated using the independent *t*-test (for normally distributed continuous variables), or the Pearson-Chi^2^-Test (for categorical variables); in comparison groups with *n* ≤ 5 cases, Fisher’s exact test was applied. (*p* < 0.05).

	Total	TXA	Non-TXA	*p*-Value
Number of patients	58	24	34	-
Age at the time of surgery (in years)	46.66 (±11.82) [27|77]	44.96 (±10.58) [32|70]	47.85 (±12.64) [27|77]	0.363
Body mass index (kg/m^2^)	27.43 (±4.63)	26.37 (±4.32)	28.18 (±4.75)	0.145
	[17.1|35.2]	[19.7|35]	[17.1|35.2]	
Gender - male - female				0.726
	10 (17.2%)	5 (20.8%)	5 (14.7%)	
	48 (82.8%)	19 (79.2%)	29 (85.3%)	
Diabetes	10 (17.2%)	3 (12.5%)	7 (20.6%)	0.499
Number of smokers	11 (19.0%)	4 (16.7%)	7 (20.6%)	1.000
Anticoagulation	4 (6.9%)	0	4 (11.8%)	0.134
History of thromboembolism	3 (5.2%)	0	3 (8.8%)	0.260

**Table 2:** Surgery-specific data. Results are given as numbers (percentage), mean (standard deviation) and [minimum|maximum]. *p*-values are calculated using the independent *t*-test (for normally distributed continuous variables), or the Pearson-Chi^2^-Test (for categorical variables); in comparison groups with *n* ≤ 5 cases, Fisher’s exact test was applied. Significant differences are shown in bold (*p* < 0.05).

	Total	TXA	No-TXA	*p*-Value
Number of patients	58	24	34	-
Indication - Post-bariatric - Esthetic				**0.041**
	40 (69.0%)	13 (54.2%)	27 (79.4%)	
	18 (31.0%)	11 (45.8%)	7 (20.6%)	
Technique - Conventional - Fleur-de-Lis				0.984
	41 (70.7%)	17 (70.8%)	24 (70.6%)	
	17 (29.3%)	7 (29.2%)	10 (29.4%)	
Additional liposuction in the abdominal region	17 (29.3%)	5 (20.8%)	12 (35.3%)	0.260
Combined procedures	12 (20.7%)	6 (25.0%)	6 (17.6%)	0.496
Rectus diastasis repair	24 (41.4%)	15 (62.5%)	9 (26.5%)	**0.006**
Weight of resection (grams)	2123 (±1676)	1713 (±1846)	2438 (±1676)	**0.024**
Time of incision to suture abdominoplasty only (hh:mm)	(*n* = 46)	(*n* = 18)	(*n* = 28)	0.338
	3:19 (±0:42)	3:11 (±0:40)	3:24 (±0:43)	
	[2:01|4:43]	[2:23|4:24]	[2:01|4:43}	
Time of incision to suture combined procedures (hh:mm)	(*n* = 12) 5:42 (±1:20) [3:46|8:40]	(*n* = 6) 5:55 (±0:55) [4:44|7:30]	(*n* = 6) 5:30 (±1:43) [3:46|7:30]	0.512

**Table 3:** Primary endpoints. Assessment of abdominal drains. Results are given as mean values (standard deviation) and [minimum|maximum]. *p*-values are calculated using the independent t-test (for normally distributed continuous variables), and the Mann–Whitney U test (for non-normally distributed continuous variables). Significant differences are shown in bold (*p* < 0.05).

	Total	TXA	No-TXA	*p*-Value
Number of patients	58	24	34	-
Length of hospital stay (days)	4.1 (±3.01)	2.79 (±1.86)	4.94 (±3.08)	**0.008**
	[1|12]	[1|7]	[1|12]	
Time to first drain removal (days)	(*n* = 53)	(*n* = 21)	(*n* = 32)	0.729
	2.17 (±1.28)	2.00 (±0.95)	2.17 (±1.28)	
	[1|7]	[1|5]	[1|7]	
Time to total drain removal (days)	(*n* = 51)	(*n* = 20)	(*n* = 31)	0.243
	5.16 (±4.16)	3.85 (±1.87)	6.00 (±4.97)	
	[1|21]	[1|7]	[1|21]	
Drainage volume in total (mL)	(*n* = 47)	(*n* = 17)	(*n* = 30)	0.352
	497 (±606)	292 (±255)	614 (±712)	
	[20|2510]	[40|1060]	[20|2510]	

**Table 4:** Postoperative complications. Results are given as number of patients (percentage). *p*-values are calculated using the Pearson-Chi^2^-Test; in comparison groups with *n* ≤ 5 cases, Fisher’s exact test was applied. Significant differences are shown in bold (*p* < 0.05).

	Total	TXA	No-TXA	*p*-Value
Number of patients	58	24	34	-
With				
- Complications in total	24 (41.4%)	6 (25.0%)	18 (52.9%)	**0.033**
- Minor complications	14 (24.1%)	4 (16.7%)	10 (29.4%)	0.356
- Major complications	10 (17.2%)	2 (8.3%)	8 (23.5%)	0.171
Seroma formation	14 (24.1%)	4 (17.4%)	10 (29.4%)	0.361
Wound healing disorder	13 (22.4%)	3 (12.5%)	10 (29.4%)	0.202
Transfusion of packed red blood cells	3 (5%)	1 (4%)	2 (6%)	1.000

**Table 5:** Postoperative laboratory parameters. Results are given as mean values in percent (standard deviation) and [minimum|maximum]. *p*-values are calculated using the independent *t*-test (for normally distributed continuous variables).

	Total	TXA	No-TXA	*p*-Value
Number of patients	54	21	33	-
Decrease in hematocrit (hct)	8.20% (±3.39)	7.66% (±3.20)	8.55% (±3.52)	0.353
	[2.4|18.1]	[2.4|13.2]	[2.7|18.1]	
Decrease in hemoglobin (hb)	2.69% (±1.09)	2.47% (±1.08)	2.82% (±1.09)	0.255
	[0.6|6.1]	[0.6|4.5]	[1.1|6.6}	

## Data Availability

The raw data supporting the conclusions of this article will be made available by the authors on request.

## Abbreviations

The following abbreviations are used in this manuscript:

TXA	Tranexamic Acid
Hb	hemoglobin
Hct	hematocrit
BMI	Body Mass Index
PTS	Progressive tension sutures
